# Effect of High-Flow Nasal Oxygen Versus Conventional Oxygen Therapy on Postoperative Atelectasis in Obese Cardiac Surgical Patients: Randomized Controlled Trial

**DOI:** 10.7759/cureus.89876

**Published:** 2025-08-12

**Authors:** Aditi Banka, Rati Prabha, Rajesh Raman, Mohammad P Khan, Sarvesh Kumar, Suhail S Siddiqui, Akshay Anand, Bhupendra Kumar

**Affiliations:** 1 Anesthesiology, Baba Raghav Das Medical College, Gorakhpur, IND; 2 Anesthesiology, King George's Medical University, Lucknow, IND; 3 Cardiovascular and Thoracic Surgery, King George's Medical University, Lucknow, IND; 4 Critical Care Medicine, King George's Medical University, Lucknow, IND; 5 Surgery, King George's Medical University, Lucknow, IND

**Keywords:** adult cardiac surgery, high-flow oxygen, lung ultrasound score, obesity, postoperative hypoxia, postoperative pulmonary complications, pulmonary atelectasis, tracheal extubation

## Abstract

Background aims

There is a high incidence of postoperative atelectasis in obese patients undergoing cardiac surgery. High-flow nasal oxygen (HFNO) therapy may decrease the incidence and severity of atelectasis in obese patients. This study aimed to compare the effects of HFNO on postoperative atelectasis with conventional oxygen therapy in obese patients after cardiac surgery with fast-track extubation.

Material and methods

This prospective, randomized, controlled, open-label study was done at a tertiary care hospital. Seventy-two patients of either gender of age 18-60 years with body mass index ≥30 kg/m^2^, scheduled to undergo elective cardiac surgery received either HFNO (group H, n=36) or conventional oxygen therapy using Hudson mask (group C, n=36) after extubation. The incidence of atelectasis was the primary outcome variable.

Results

In group H, the incidence of atelectasis was lower at 24 (63.9% versus 88.9%, p=0.025) and 72 (58.33% versus 86.11%, p=0.017) hours after extubation. The severity of atelectasis and shunt fraction were lower in group H at 24 and 72 hours. The partial pressure of oxygen (PO_2_) and the ratio of PO_2_ and fraction of inspired oxygen were higher with HFNO use. The duration of oxygen therapy and intensive care unit stay was shorter in the HFNO group.

Conclusion

It is concluded that HFNO is superior to conventional oxygen therapy for oxygenation after extubating obese patients undergoing cardiac surgery, resulting in reduced atelectasis and improved oxygenation.

## Introduction

Patients who undergo cardiac surgery face numerous pulmonary complications in the postoperative period, including atelectasis, pneumothorax, hemothorax, pleural effusion, pulmonary edema, pneumonia, and phrenic nerve palsy [1]. These complications increase the morbidity, mortality, and treatment costs of cardiac surgical patients. Up to 90% of post-cardiac surgery cases have atelectasis [2]. Atelectasis impairs pulmonary gas exchange and causes hypoxemia [1]. Atelectasis also contributes to lung injury by impairing pulmonary compliance, inducing local inflammation and immune dysregulation [3]. Obesity increases the incidence and severity of postoperative pulmonary complications, including atelectasis [4].

Strategies to prevent postoperative atelectasis include lung recruitment maneuver, non-invasive ventilation, adequate analgesia, respiratory physiotherapy, and early mobilization [4]. In various studies, high-flow nasal oxygenation (HFNO) application after tracheal extubation has reduced postoperative atelectasis [5,6]. In a trial conducted on patients with obesity undergoing cardiac surgical procedures, HFNO use was associated with significantly better oxygenation and lower atelectasis severity than conventional oxygen therapy [6]. However, the atelectasis severity was not the primary outcome variable in the study, and its incidence was not reported. In another study conducted on patients with morbid obesity undergoing bariatric surgery, HFNO use reduced the incidence of atelectasis [7].

This trial aimed to compare the effects of HFNO and conventional oxygen therapy on postoperative atelectasis in obese patients after cardiac surgery with fast-track extubation. It was hypothesized that compared to conventional oxygen therapy, HFNO use after tracheal extubation of obese patients undergoing fast-track cardiac surgery reduces postoperative atelectasis. The primary objective of the trial was to compare the effects of the above two oxygenation methods on the incidence of postoperative atelectasis in patients undergoing cardiac surgical procedures. The secondary objectives were to compare the severity of atelectasis, oxygenation, shunt fraction (SF), duration of oxygen therapy and intensive care unit (ICU) stay, dyspnea, and complications between the two oxygen delivery methods in patients undergoing cardiac surgical procedures.

## Materials and methods

This prospective, randomized, controlled, open-label, and parallel-arm trial was conducted between 31^st^ May to 2^nd^ December 2022 in a post-surgical ICU of a university hospital after permission from the ethics committee (King George's University Institutional Ethics Committee, approval No. 1039/ethics/2021, dated 13/8/2021) and registration with Clinical Trial Registry of India (registration number: CTRI/2022/05/042678). The trial followed the guidelines in the Declaration of Helsinki, 2013. Our trial included patients of American Society of Anesthesiologists (ASA) physical status III and IV of either gender, aged 18 to 60 years, with body mass index (BMI) ≥30 kg/m^2^, planned for elective cardiac surgery with fast-track extubation. The trial excluded patients with heart failure, severe pulmonary hypertension, preoperative pulmonary disease, on vasopressor support, having contraindications to HFNO, including blocked nasal passage/choanal atresia, trauma/surgery of the nasopharynx, and refusal to give consent.

Informed and written consent was taken from the recruited participants. Participant recruitment and allocation concealment were carried out using a sequentially numbered opaque sealed envelope technique by the second and fifth authors. The randomization sequence was created using computer-generated random numbers by the fourth author. The participants were allocated one of these two groups: Group C (n=36) - patients were kept on conventional oxygen therapy after extubation with 4-8 liters/minute oxygen flow to maintain arterial oxygen saturation (SpO_2_) greater than 94% using a Hudson mask; and group H (n=36) - patients were kept on HFNO (Inspired O2FLO, Vincent Medical Manufacturing, Hang Fung, Hong Kong) after extubation for a minimum of 12 hours with oxygen flow 30-60 liters/minute and concentration (FiO_2_) titrated to maintain SpO_2_ greater than 94%.

An intravenous line was secured in the operating theatre. For monitoring, a pulse oximeter, invasive blood pressure (using the radial artery), and electrocardiography were applied. Oxygen supplementation with a Hudson mask was done for all the patients. Induction of general anesthesia was carried out using intravenous (IV) injections of midazolam (0.05-0.08 mg/kg) and fentanyl (2-5 microgram/kg), followed by injection etomidate (titrated doses between 0.2-0.3 mg/kg), and injection vecuronium (0.08-0.1 mg/kg). Maintenance of anesthesia was achieved using a mixture of oxygen, air, and sevoflurane (0.5-1 MAC) with boluses of injection fentanyl (1-2 microgram/kg) and injection vecuronium (0.04-0.06 mg/kg) IV. The patients were ventilated with a tidal volume of 6-8 ml/kg and positive end-expiratory pressure (PEEP) of 5-8 cm H_2_O. The FiO_2_ was 40-60%, titrated to maintain SpO_2_ more than 94%. During the cardiopulmonary bypass, mechanical ventilation was stopped. At the end of surgery, lung recruitment was done by increasing the airway pressures to 40 cm H_2_O for 30-40 seconds. After the surgery ended, the patients were shifted to a postoperative intensive care unit (ICU) without tracheal extubation.

**Figure 1:**
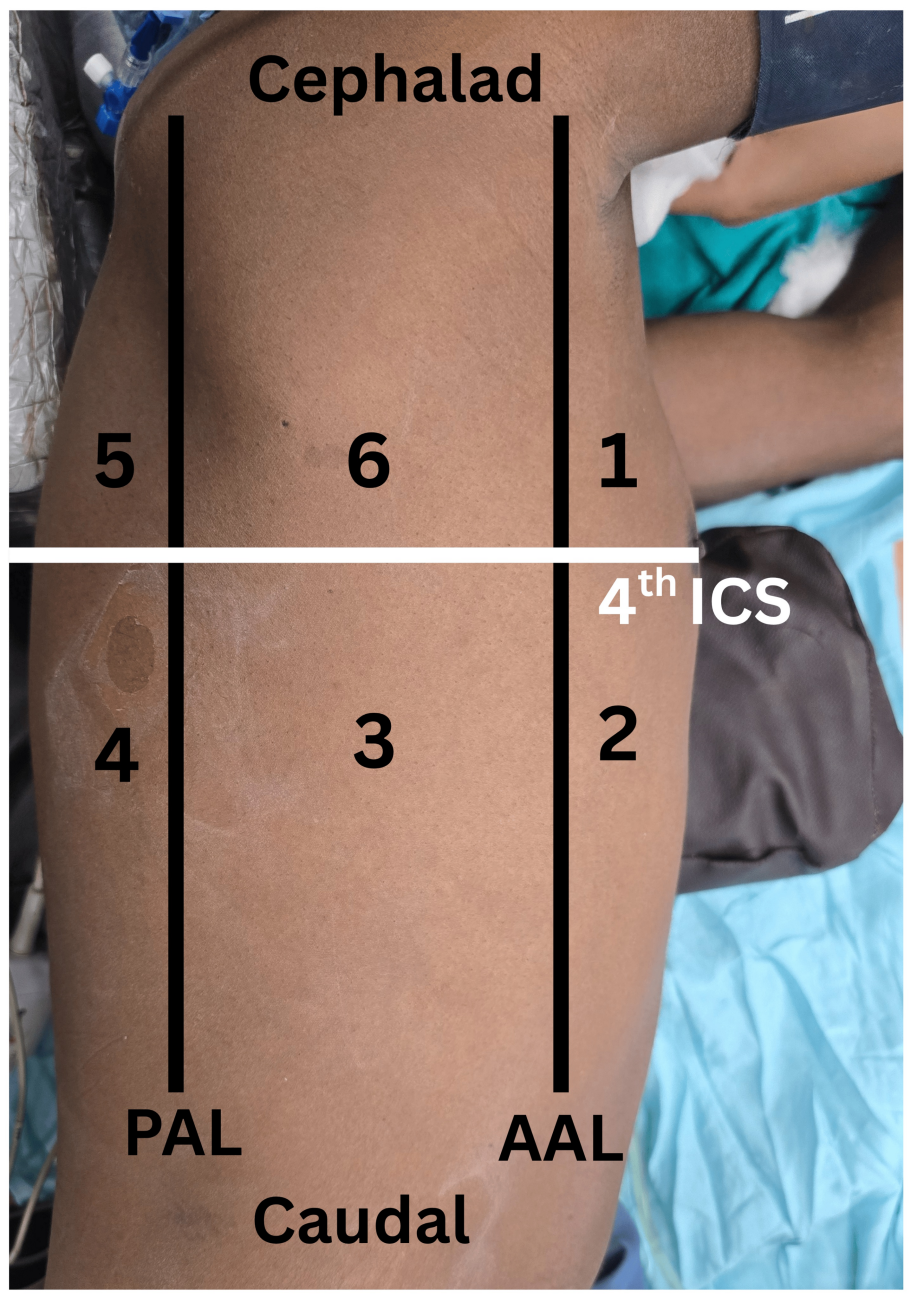
Surface landmarks dividing each hemithorax into six zones for assessment of atelectasis using lung ultrasound. ICS: intercostal space, PAL: posterior axillary line. AAL: anterior axillary line.

**Figure 2:**
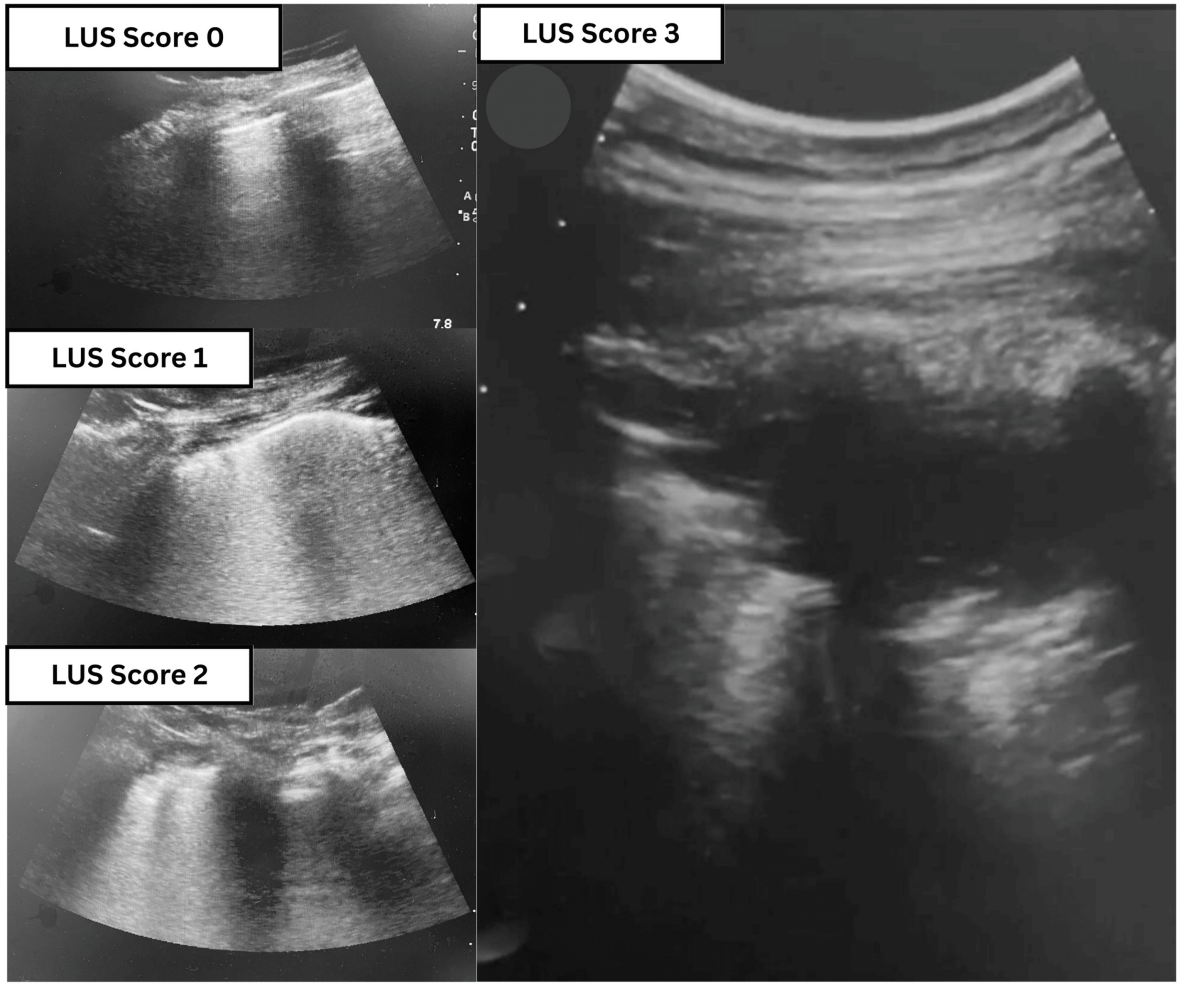
Classification of lung atelectasis severity using lung ultrasound. Severity of atelectasis using Lung ultrasound.

Atelectasis was assessed in the postoperative ICU using the lung ultrasound score (LUS) and the radiological atelectasis score (RAS). For LUS, images were acquired with a Mylab Seven (Esaote SpA, Genoa, Italy) ultrasound machine using a 1-8-megahertz curvilinear probe. Each hemithorax was subdivided into six zones (Figure 1) by anterior and posterior axillary lines and one transverse line at the fourth intercostal space level [7]. The following LUS was used to grade atelectasis in each scanned zone: 0 = no aeration loss with normal lung, smooth pleura, and A-lines; 1 = moderate aeration impairment with fewer than three discrete B-lines arising from a smooth pleura; 2 = severe aeration impairment with interrupted pleura and confluent B-lines; 3 = total absence of aeration with a tissue-like pattern or sub-pleural consolidation (Figure 2) [8]. Anteroposterior chest x-ray was used for the assessment of postoperative atelectasis using RAS: 1 = clear lung fields; 2 = slight infiltration or plate-like atelectasis; 3 = partial atelectasis; 4 = unilateral lobar atelectasis; 5 = bilateral lobar atelectasis [9].

A score of LUS≥1 or RAS≥2 was defined as the presence of atelectasis. The scoring was done by the first and third authors. In case of disagreement between the scores, the help of the fourth author was taken for the scoring. The three authors have at least five years of experience in lung ultrasound. Individual LUS scores of each zone were added to calculate the severity of atelectasis. Presence and severity of atelectasis, partial pressures of carbon dioxide (PCO_2_) and oxygen (PaO_2_) in blood, shunt fraction (SF), ratio of PaO_2_ and FiO_2_ (P/F ratio) were recorded at the time of extubation and at 24 and 72 hours after extubation. The heart rate (HR), SpO_2_, severity of dyspnea using the modified Borg scale, respiratory rate, pain using a ten-centimeter visual analog scale, and mean arterial pressure (MAP) were assessed every two hours till 12 hours after extubation, then on the first, second, and third postoperative days [10]. The incidence of atelectasis was the primary outcome variable, while respiratory rate, SpO_2_, severity of atelectasis, SF, P/F ratio, PCO_2_, dyspnea, mean arterial pressure, duration of oxygen and ICU admission, and complications were the secondary outcomes.

SF was measured using the following equation [11]:

$SF = \frac{C_{c}O_{2} - C_{a}O_{2}}{C_{c}O_{2} - C_{v}O_{2}}$

Where:

$C_{a}O_{2} = (1.36 \times S_{a}O_{2} \times \text{Hemoglobin}) + (0.0031 \times P_{a}O_{2})$

$C_{c}O_{2} = \left( 1.36 \times \text{Hemoglobin} \right) + 0.0031 \times \left\{ F_{i}O_{2} \left( P_{B} - P_{H_{2}O} \right) - \frac{P_{a}CO_{2}}{RQ} \right\}$

$C_{v}O_{2} = \left( 1.36 \times S_{v}O_{2} \times \text{Hemoglobin} \right) + \left( 0.0031 \times P_{v}O_{2} \right)$

In the equations, PB = atmospheric pressure (760 mmHg); PH₂O = saturated vapor pressure at 37°C (= 47 mmHg); RQ = respiratory quotient (= 0.8); C_c_O₂, C_a_O₂, C_v_O₂ = oxygen content of pulmonary capillary, systemic artery, and mixed venous blood respectively; S_a_O₂, S_v_O₂ = the arterial and central venous oxygen saturation respectively; P_v_O₂ = central venous partial pressure of oxygen. For C_v_O_2_, blood from the right atrium was used instead of the usual pulmonary artery.

Our study had a power of 80% and a type I error of 5%. To detect a clinically meaningful 30% difference in the incidence of atelectasis with a 90% incidence of atelectasis in the control group, at least 32 participants were required in each group [2]. Thirty-six patients were enrolled in each group to account for lost data and patient exclusions. Students' t-test, Mann-Whitney U test, and Fisher’s exact test were used for analyzing continuous, ordinal, and nominal data, respectively. All the tests were judged to have achieved statistical significance when the two-sided p-value was <0.05. Values are shown as mean with standard deviation, median (interquartile range), and number (percentages). 95% confidence intervals are also presented for mean and median values. There were no modifications to study methods after the start of the trial.

## Results

**Figure 3:**
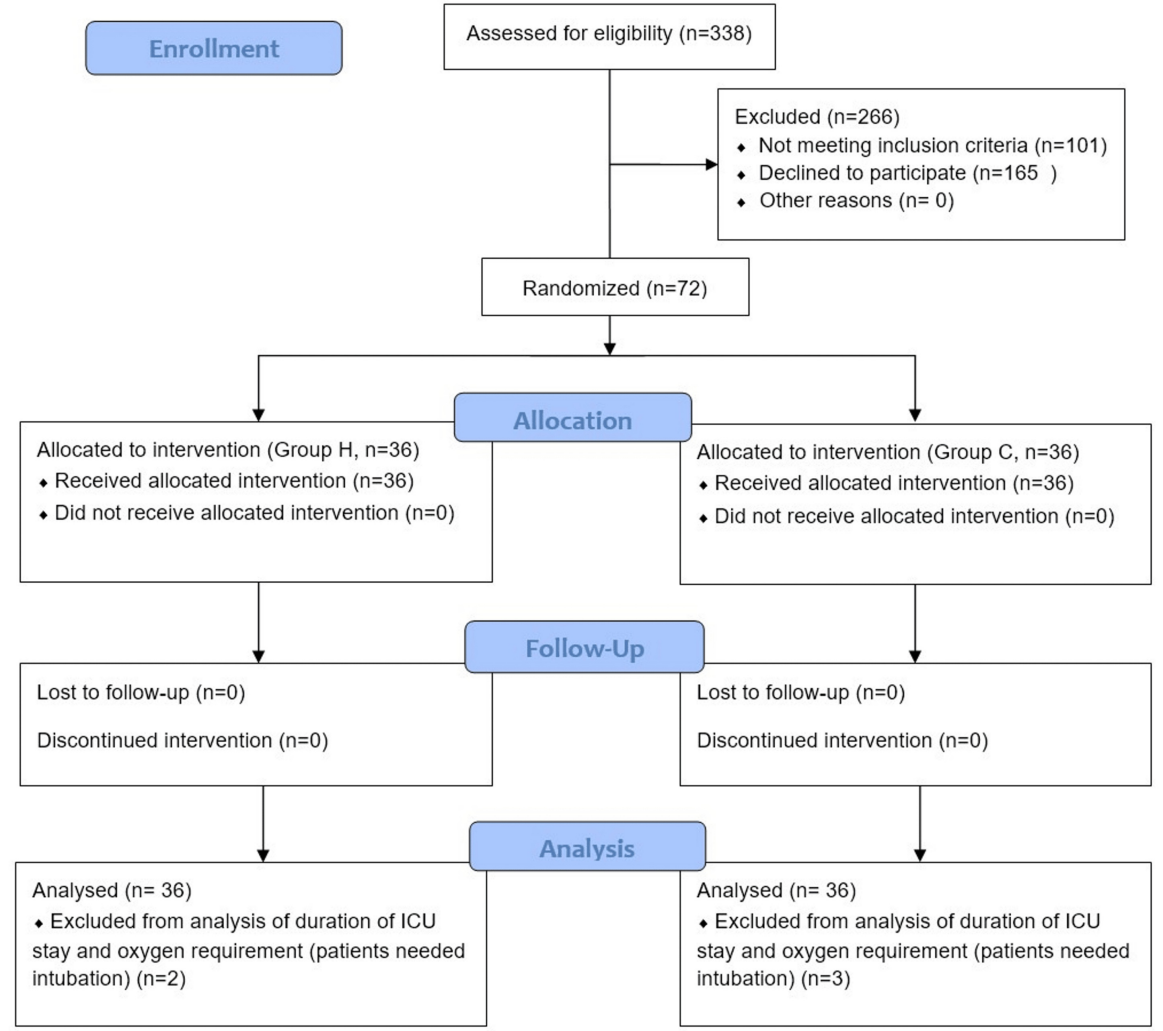
CONSORT diagram depicting the flow of patients in the study. CONSORT: CONsolidated Standards Of Reporting Trials

**Figure 4:**
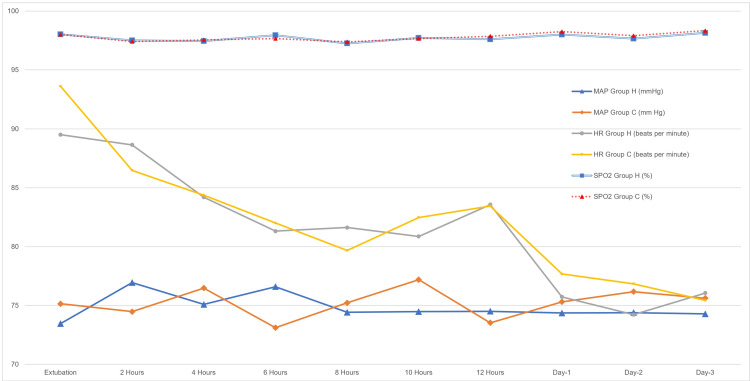
Comparison of heart rate (HR), mean arterial pressure MAP), and arterial oxygen saturation (SpO2) between the groups.

**Figure 5:**
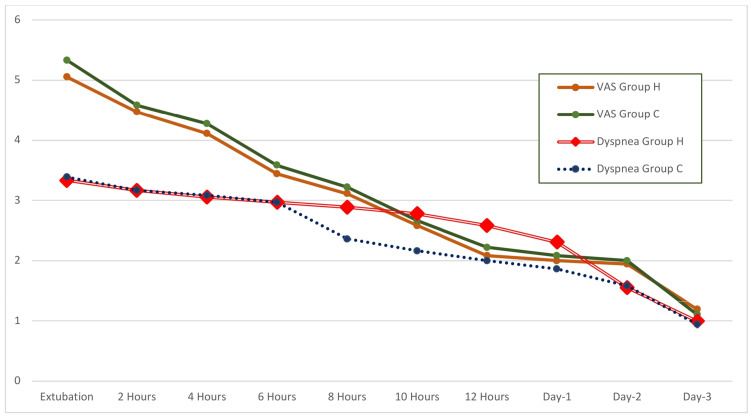
Comparison of postoperative pain and dyspnea between the groups Pain is shown using a ten-centimeter visual analog scale and dyspnea is shown on modified Borg scale. VAS: Visual analog scale.

**Figure 6:**
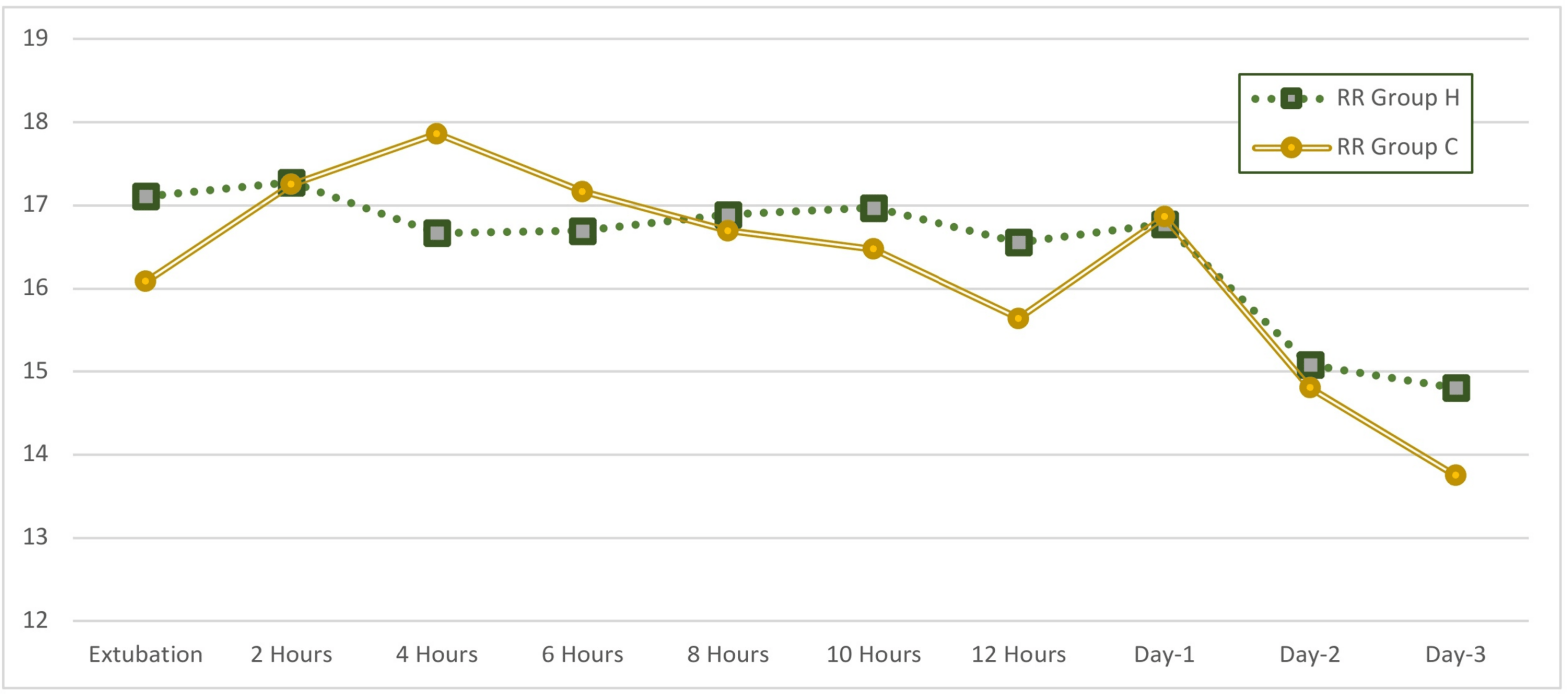
Comparison of respiratory rate between the two groups. Respiratory rate is shown in rate per minute. RR: respiratory rate.

A flowchart representing the flow of patients through the trial is depicted in Figure 3. Two patients in group H and three in group C needed reintubation after 72 hours. They were excluded from the analysis of the duration of oxygen therapy and ICU stay. The difference in baseline data was statistically non-significant, as shown in Table 1. Atelectasis and arterial blood gas parameters are summarized in Table 2. Incidence and severity of atelectasis were statistically lower in group H at 24 and 72 hours after extubation. In group H, SF was lower, while the P/F ratio and PO_2_ were statistically higher at 24 and 72 hours. Duration of oxygen therapy and ICU stay were significantly lower in group H (Table 3). HR, MAP, and SpO_2_ were statistically similar at all the time points of observation (Figure 4). The differences observed in postoperative pain and dyspnea were not statistically significant (Figure 5). The respiratory rate in the postoperative period was statistically similar (Figure 6). Complications are summarized in Table 4. Complications were statistically similar between the two groups.

**Table 1:** Baseline and demographic characteristics of the patients Data are represented as mean±standard deviation or number(percentage); cm: centimeters, Kg: kilograms, BMI: body mass index, M: male, F: female, ASA: American Society of Anesthesiologists physical status. CABG: coronary artery bypass grafting, ASD: atrial septal defect, VSD: ventricular septal defect.

	Group H (n=36)	Group C (n=36)	p
Age (years)	40.67±14.62	39.47±14.83	0.732
Height (cm)	157.17±8.47	160.42±6.65	0.075
Weight (Kg)	79.25±8.38	82.64±7.67	0.078
BMI (Kg/m^2^)	32.05±1.90	32.06±1.59	0.979
Gender (M/F)	20(55.56%)/16 (44.4%)	15(41.7%)/21(58.33%)	0.346
ASA (III/IV)	20(55.56%)/16(44.44%)	24(66.67%)/12(33.33%)	0.469
Type of surgery			0.995
Mitral valve replacement	6(16.67%)	4(11.11%)	
Aortic valve replacement	4(11.11%)	4(11.11%)	
CABG	15(14.67%)	16(44.44%)	
ASD repair	2(5.56%)	2(5.56%)	
VSD repair	3(8.33%)	3(8.33%)	
Double valve replacement	6(16.67%)	7(19.44%)	

**Table 2:** Clinical outcomes of the patients in two groups Data are represented as mean ± standard deviation, median(interquartile range) with 95% confidence intervals, or number(percentage); *: statistically significant (p<0.05), LUS: lung ultrasound score, RAS: radiological atelectasis score, PaO_2_: partial pressure of oxygen in arterial blood, PaCO_2_: partial pressure of carbon dioxide in arterial blood, P/F: ratio of PO_2_ and fraction of inspired oxygen, CI: confidence interval.

		Group H (n=36)	95% CI	Group C (n=36)	95% CI	p	Effect size
Atelectasis incidence (%)	After extubation	32(88.89%)		33(91.67%)		1.000	NA
	24-hours	23(63.89%)		32(88.89%)		0.025*	
	72-hours	21(58.33%)		31(86.11%)		0.017*	
Atelectasis severity using LUS	After extubation	10.00(8.00-12.00)	8.00-12.00	12.00(9.00-14.25)	10.00-14.00	0.078	
	24-hours	9.50(0.00-12)	0.00-12.00	12(10-14.00)	10.00-14.00	0.008*	
	72-hours	8.00(0.00-8.50)	0.00-8.00	9.50(6.00-12.00)	7.00-10.00	0.021*	
Atelectasis severity using RAS	After extubation	1.50(1.00-2.00)	1.00-2.00	2.00(1.00-3.00)	1.00-3.00	0.078	
	24-hours	1.00(0.00-2.00)	0.00-2.00	2.00(1.00-3.00)	1.00-3.00	0.037*	
	72-hours	1.00(0.00-1.00)	0.00-1.00	1.00(0.75-2.00)	1.00-2.00	0.038*	
Shunt fraction (%)	After extubation	11.62±2.11	10.90-12.33	11.80±2.17	11.06-12.53	0.721	0.084
	24-hours	10.30±1.38	9.84-10.78	11.15±2.10	10.44-11.86	0.048*	0.478
	72-hours	9.36±1.60	8.82-9.90	10.39±1.66	9.83-10.95	0.009*	0.632
P/F	After extubation	271.44±32.38	260.49-282.40	260.35±26.61	251.35-269.64	0.117	-0.374
	24-hours	303.35±26.00	294.55-312.14	272.97±34.79	261.20-284.75	<0.001*	-0.989
	72-hours	342.27±20.97	335.18-349.36	327.22±27.31	317.98-336.46	0.011*	-0.618
PaO_2 _(mm Hg)	After extubation	125.83±31.23	115.27-136.40	117.50±14.56	112.58-122.43	0.153	-0.342
	24-hours	120.40±16.98	114.66-126.15	105.29±13.72	100.65-109.93	<0.001*	-0.979
	72-hours	76.59±5.03	74.88-78.29	70.92±7.98	68.22-73.62	0.001*	-0.849
PaCO_2 _(mm Hg)	After extubation	36.11±3.71	34.856-37.37	36.21±3.19	35.13-37.29	0.905	-0.028
	24-hours	37.33±3.56	36.12-38.53	37.59±2.34	36.80-38.38	0.712	-0.087
	72-hours	35.71±2.89	34.73-36.69	36.89±3.17	35.81-37.96	0.104	0.389

**Table 3:** Comparison of duration of oxygen therapy and ICU stay Data are represented as mean ± standard deviation with 95% confidence intervals; *: statistically significant (p<0.05), ICU: intensive care unit, CI: confidence intervals.

	Group H (n=34)	95% CI	Group C (n=33)	95% CI	p	Effect size
Duration of oxygen therapy (hours)	38.65±5.59	36.70-40.60	50.15±5.51	48.20-52.10	<0.001*	2.072
Duration of ICU stay (hours)	61.73±4.80	60.03-63.43	64.97±5.89	62.92-67.02	0.016*	0.603

**Table 4:** Comparison of incidence of complications Data are represented as number(percentage).

	Group H (n=36)	Group C (n=36)	p
Heart Failure	1(2.78%)	1(2.78%)	1.000
Pneumonia	2(5.56%)	1(2.78%)	1.000
Nasal bleed	1(2.78%)	1(2.78%)	1.000
Reintubation rate	2(5.56%)	3(8.33%)	1.000

## Discussion

In this trial, we compared the effects of HFNO and conventional oxygen therapy on postoperative atelectasis in obese patients undergoing elective cardiac surgery followed by fast-track tracheal extubation. It was observed that using HFNO after extubation reduced the incidence and severity of atelectasis. Also, SF was lower, and oxygenation (P/F ratio and PO_2_) was better with HFNO use. The duration of oxygen therapy and ICU stay was lower with HFNO use. PaCO_2_, HR, MAP, respiratory rate, SpO_2_, dyspnea, and complications were similar in both groups.

Both cardiac surgery and obesity have a high incidence of atelectasis in the postoperative period [2-4,6]. Postoperative atelectasis after cardiac surgery develops because of general anesthesia, apnea during cardiopulmonary bypass, manual manipulation of lungs, internal mammary artery harvesting, pleurotomy in the intraoperative period, and pain during the postoperative period. The chief mechanisms responsible for atelectasis after cardiac surgery are lung tissue compression, alveolar air absorption, and lung surfactant disruption [1,5]. The high incidence of atelectasis after cardiac surgery is compounded by reduced functional residual capacity (FRC) and reduced lung compliance in obesity [4,12,13]. Patients with obesity have a greater risk of postoperative atelectasis due to reduced FRC and raised intra-abdominal pressures [14]. In addition, atelectasis persists for a longer duration in obese patients [4]. Avoiding respiratory complications, including atelectasis, is especially important in fast-track cardiac surgery, as they can result in delayed extubation or need for reintubation, causing fast-track extubation failure [15].

In the current trial, atelectasis incidence and severity were significantly reduced with post-extubation use of HFNO. We could not find any study evaluating the effects of HFNO on the incidence of atelectasis following cardiac surgery. The severity of atelectasis, assessed using RAS, was reduced with HFNO use in obese cardiac surgical patients at 12, 24, 36, and 48 postoperative hours [6]. In non-obese cardiac surgical patients also, the severity of atelectasis was reduced in patients receiving HFNO and pecto-intercostal block [5]. The control group in this study received conventional oxygen therapy without the nerve block for pain relief. However, in another study on 155 obese patients undergoing cardiac surgery, HFNO did not improve the severity of postoperative atelectasis on the first and fifth postoperative days [2]. The authors in the study assessed the severity of atelectasis using only RAS. Ultrasound scoring for atelectasis was not used. This may have caused a lower detection rate of atelectasis incidence than our study, as we used both RAS and LUS for detecting atelectasis. Another reason for the difference in the outcomes may be the difference in the duration of postoperative HFNO use. The authors used HFNO for eight hours only, while we used HFNO for a minimum of 12 hours. As the incidence and severity of atelectasis can be higher in the subset of patients undergoing cardiac surgery with obesity, HFNO use is more advantageous than in other situations [6].

HFNO is designed for the management of spontaneously breathing people with respiratory disorders. The use of HFNO supports respiration, reduces atelectasis and postoperative pulmonary complications by assuring a constant FiO_2_ with high flows of adequately humidified air, washout, and improvement of gas mixing in anatomic dead space, improving lung compliance [16-20]. The PEEP generated by the high flow of gases leads to the recruitment of atelectatic alveoli and counterbalances auto-PEEP, decreasing the work of breathing [16,17].

The shunt fraction was lower in patients treated with HFNO in the postoperative period. Atelectasis produces an intrapulmonary shunt as the collapsed, non-ventilated alveoli are still perfused [4]. This increases the SF and decreases the oxygenation [21-23]. The re-expansion of atelectatic alveoli due to PEEP generated by HFNO reduces the intrapulmonary shunt and improves oxygenation [16]. In the current study, this resulted in a higher P/F ratio and PO_2_ in the patients receiving HFNO. Despite significantly higher PO2 in the HFNO group, the difference in SPO_2_ was insignificant in our study. The non-linear relationship between SpO2 and PO2 explains this, as PO_2_ may continue to rise despite a small increase in SpO_2_, especially in the flat part of the oxygen-hemoglobin dissociation curve [24,25]. Better oxygenation may be responsible for the reduced duration of oxygen therapy and ICU stay in patients with HFNO. The use of HFNO had complications similar to those of conventional oxygen therapy in our study, suggesting a similar safety profile.

Apart from being a monocentric study, the other limitation of the current trial is that it is an open-label trial, as the study design did not allow blinding of the participants and observers. This may have introduced bias in the trial. Second, preoperative SF was not known in the patients, as central venous access was acquired after the induction of anesthesia.

## Conclusions

We studied the effects of HFNO on postoperative atelectasis and compared it with conventional oxygen therapy in obese patients after cardiac surgery with fast-track extubation. It was observed that using HFNO after extubation reduced the incidence and severity of atelectasis. It is concluded that HFNO is superior to conventional oxygen therapy for oxygenation after extubating obese patients undergoing elective cardiac surgery with fast-track extubation, resulting in reduced atelectasis and improved oxygenation.
